# Selection and validation of reference genes for quantitative real-time PCR during the developmental stages of seeds in *Sophora davidii*

**DOI:** 10.3389/fpls.2025.1485586

**Published:** 2025-05-16

**Authors:** Jingjing Li, Shanrong Han, Zongren Xu, Bin Deng, Na Zheng, Yaqiong Su, Ziyao Qiao, Yun Yang, Hong Zhang, Zongsuo Liang, Jing Liu, Shuai Liu

**Affiliations:** ^1^ Key Laboratory of Resource Biology and Biotechnology in Western China, Ministry of Education/College of Life Science, Northwest University, Xi’an, Shaanxi, China; ^2^ Institute of Chinese Materia Medica, Shaanxi Academy of Traditional Chinese Medicine, Xi’an, Shaanxi, China; ^3^ School of Pharmacy, Guizhou University of Traditional Chinese Medicine, Guiyang, China

**Keywords:** *Sophora davidii*, reference gene, development stages of seeds, stability, normalization

## Abstract

**Introduction:**

Quinolizidine alkaloids, such as matrine and sophocarpine, enriched in *Sophora davidii* seeds, demonstrate notable anticancer properties. However, the biosynthetic pathway of these alkaloids remains incompletely elucidated, and the expression patterns of key enzyme genes involved in this pathway require further investigation. Quantitative real-time PCR (qRT-PCR) serves as a highly sensitive method for gene expression analysis, yet selecting appropriate reference genes is crucial to ensure the accuracy and reliability of results.

**Methods:**

Ten candidate reference genes (*18S, ACT13, RL15B, RL74, RLA2, RL182, RL291, EF1-α, EF1G*, and *YLS8*) were evaluated for their expression stability in *Sophora davidii* seeds collected at five distinct developmental stages post-flowering, characterized by significant morphological changes. Five computational tools—GeNorm, NormFinder, BestKeeper, ΔCt, and RefFinder—were employed to comprehensively analyze the stability of these genes.

**Results:**

Among the candidate genes, *EF1G* and *RL291* exhibited the highest expression stability, whereas *RL182* proved unsuitable as a reference gene. Validation experiments confirmed that normalization using stable reference genes (e.g., *EF1G* and *RL291*) yielded accurate quantification of target gene expression.

**Discussion:**

This study identifies *EF1G* and *RL291* as optimal reference genes for qRT-PCR analysis during Sophora davidii seed development, addressing a critical methodological gap in alkaloid biosynthesis research. These findings underscore the necessity of rigorous reference gene validation to ensure reliable gene expression data. The results advance our understanding of quinolizidine alkaloid biosynthesis and highlight the broader importance of reference gene selection in plant molecular studies.

## Introduction

*Sophora davidii* (*S. davidii*), also known as *Sophora viciifolia* (Xu et al., 2023), is commonly found in hillside, wayside, or bush habitats at elevations spanning from 2000 to 3500 meters (Tai et al., 2011). It has a wide distribution across China, with prominent occurrences in Guizhou, Yunnan, and the northwestern regions. This plant can thrive in diverse environments, highlighting its significant ecological adaptability and diversity (Wang and Yilihamu, 2021). As a pioneer shrub species, it plays a crucial role in protecting rangelands from wind erosion and is of great significance in maintaining the stability of desert ecosystems.

In traditional Chinese medicine, the roots, leaves, flowers, and seeds of *S. davidii* have long been utilized to treat internal heat, sore throats, and swelling (Li et al., 2009). This plant is rich in alkaloids such as matrine (MT), oxymatrine (OMT), and sophoridine (SRI), which are well-known for their medicinal properties (Zhang et al., 2021), including antitumor (Li et al., 2020; Rashid et al., 2019; Bunsupa et al., 2012), antiviral (Zhang Y. B. et al., 2018; Zou et al., 2020; Zhang T. et al., 2024), anti-inflammatory, and antibacterial effects (Wink, 2019; Chen et al., 2005; Wang et al., 2021, 2019; Li et al., 2023; Zhang B. C. et al., 2018; Lin et al., 2019). Furthermore, with its profuse flowers and ample nectar, *S. davidii* serves as an important honey-producing plant in the spring, yielding high-quality honey (Wang et al., 2023).

Currently, molecular biology research on *S. davidii* is still in its early stages, and there is relatively limited research on the identification of reference genes for gene expression analysis. Understanding gene expression changes is a critical initial step in studying gene function. To compare gene expressions among different samples, it is essential to first normalize the data using a common factor (Yim et al., 2015). With the continuous progress of related research, the identification of stable reference genes for the study of gene expressions in *S. davidii* has become increasingly important. To further explore the synthesis mechanism of active compounds in *S. davidii*, an analysis of the expressions of relevant genes is required.

Quantitative real-time polymerase chain reaction (qRT-PCR) is widely employed in molecular biology and medical research to measure gene expression levels due to its high sensitivity, specificity, accuracy, and speed (Zhao X. D. et al., 2022; Freitas et al., 2021; Zhao J. M. et al., 2022; Fazzina et al., 2024). The reliability of qRT-PCR results depends on the selection of stable reference genes to standardize target gene data (Huggett et al., 2005). However, the expressions of reference genes may vary depending on the tissue or material being studied, which can affect the interpretation of the data. Therefore, selecting reference genes with stable expressions is crucial for ensuring accurate gene expression analysis (Lucho et al., 2018). Studies have shown that no single reference gene can maintain stable in all tissues and under all experimental conditions. The stability of reference gene expression typically depends on specific tissues or experimental environments (Remans et al., 2014; Chen et al., 2020). Previous studies have shown that there are significant differences in the expression patterns of housekeeping genes based on genetic profiles, cell types, and experimental parameters (Panina et al., 2018). Under different conditions, the use of inappropriate reference genes can lead to inaccurate expression profiles of target genes. Currently, suitable reference genes have been identified for plants such as *Codonopsis pilosula* (Liang et al., 2020), *Artemisia* sp*haerocephala* (Hu et al., 2018), *Kengyilia melanthera* (Zhao J. M. et al., 2022), *Isatis indigotica* Fort (Li T. et al., 2017), and *Dalbergia odorifera* (Meng et al., 2019). Changes in gene expression levels are direct biomarkers for evaluating an organism’s response to environmental changes (Kubista et al., 2006). In theory, the expression levels of reference genes should be consistent across all samples. However, practical studies have demonstrated that no reference gene can be stably expressed in all tissues and under all conditions. Stable expressions of specific reference genes is often only observed in specific tissues or under specific conditions (Wang and Bhullar, 2021; Li et al., 2019; Zhou et al., 2019; Zheng et al., 2018). qRT-PCR not only allows for the precise quantification of target gene expressions under controlled conditions but also provides valuable insights into the gene regulatory mechanisms underlying the biosynthesis of active compounds.

RNA sequencing (RNA-seq) has emerged as a vital tool for comprehensive gene expression analysis. Leveraging the Illumina sequencing platform, RNA-seq can detect all mRNA transcripts in specific tissues or cells at specific time points, which is of great significance for understanding gene function and structure (Wang et al., 2009). This approach plays a crucial role in uncovering the developmental processes and disease mechanisms of organisms (Wang et al., 2009; Majewski and Pastinen, 2011). RNA-seq is widely employed to identify genes with differential expressions under various experimental conditions. Genes that exhibit stable expressions across different tissues or conditions are potential candidates for reference genes (Stanton et al., 2017; Bowen et al., 2014). However, to date, no research has identified genes with stable expressions in *S. davidii*, and there is limited information regarding the identification and validation of qRT-PCR normalized reference genes in *S. davidii*.

In earlier research, we discovered that the alkaloid content in the seeds of *S. davidii* was higher than that in other tissues (Xu et al., 2024). In this study, we collected seeds from different developmental stages of *S. davidii*. 10 commonly used housekeeping genes were selected, namely 18S ribosomal RNA (*18S*), actin-13 (*ACT13*), ribosomal protein L15-2 (*RL15B*), ribosomal protein L7-4 (*RL74*), acidic ribosomal protein P2 (*RLA2*), ribosomal protein L18-2 (*RL182*), ribosomal protein L29-1 (*RL291*), elongation factor 1-α (*EF1-α*), eukaryotic translation elongation factor 1 gamma (*EF1G*), and thioredoxin-like protein YLS8 (*YLS8*). At different stages of seed development, statistical methods such as ΔCT, GeNorm, NormFinder, BestKeeper, and RefFinder were utilized to evaluate these genes.

Additionally, we validated the selected reference genes by analyzing Lysine decarboxylase (LDC), which is involved in alkaloid biosynthesis (Mancinotti et al., 2022), along with nine genes associated with seed size and lipid synthesis. These nine genes include fatty acid desaturase (*FAD*) (Al Amin et al., 2019), transcription factor MYB73 (*MYB73*) (Liu et al., 2014), B3 domain transcription factor LEC2 (*LEC*) (Manan et al., 2017), transcription factor encoding (*NYFA*) (Lu et al., 2016), the enzyme encoding the rate-limiting step of gibberellin biosynthesis (*GA20OX*) (Lu et al., 2016), phosphatase 2C (*PP2C*) (Lu et al., 2017), cytochrome P450 78A (*CYP*) (Wang et al., 2015), G-patch domain-containing protein (*GPAT*) (Hirawake-Mogi et al., 2021) and plasma membrane intrinsic protein (*PIP*) (Lu et al., 2018). The findings of this study are expected to provide valuable references for elucidating the molecular mechanisms governing the development of *S. davidii*.

## Materials and methods

### Plant materials

The seeds of *S. davidii* were collected near Longshan Park in Yijun County, Tongchuan City, Shaanxi Province (35°23’2.9292”N, 109°6’40.7448”E). The relevant samples were archived at the Shanxi Academy of Traditional Chinese Medicine. *S. davidii* samples were harvested from June to September 2023 and categorized into five groups according to the maturity status of the fruits. The S1 stage of *S. davidii* is shortly after flowering. At this time, the pods are starting to develop, and the seeds are still small and underdeveloped. We collected seeds at the S1 stage during this particular growth phase. The S2-stage samples were collected 22 days after the collection of S1-stage seeds. By this time, the pods had slightly enlarged. The S3-stage seeds were harvested 34 days after the S1 stage, when the seeds began to swell. The S4-stage samples were collected 49 days after the S1 stage. During this period, the seeds in the pods showed a significant increase in size and became plump. The S5-stage samples were collected 64 days after the S1 stage. At this time, the pods were nearly dry, and the seeds were close to full maturity (**Supplementary Figure S1**). After harvesting, we removed the pod skins to extract the seeds. The seeds at each developmental stage were divided into three equal portions and stored under deep-freezing conditions at -80°C until RNA extraction was conducted.

### Selection of candidate reference genes

Candidate reference genes are commonly employed as housekeeping genes in both model and non-model plant species (Hu et al., 2022; Ferreira et al., 2023; Schmidt and Delaney, 2010; Radonić et al., 2004; Li J. T. et al., 2017). In this study, we initially identified genes that have been validated to exhibit stable expressions in plants through a comprehensive literature review. Subsequently, based on RNA-seq data, we selected co-expressed genes with FPKM values showing a difference of less than 2 across different samples. Finally, from common housekeeping genes, we chose 10 candidate reference genes, namely *18S*, *ACT13*, *RL15B*, *RL74*, *RLA2*, *RL182*, *RL291*, *EF-1α*, *EF1G*, and *YLS8*. We used PRIMER PREMIER 5.0 software (San Francisco, California, USA) or primer design. The primers were synthesized by Tsingke (Beijing,China). A standard curve was generated by diluting the cDNA four-fold. Based on this curve, we determined the amplification efficiency (E) and correlation coefficient (R^2^) of the primers. The amplification efficiency of each primer pair was calculated according to the slope of the linear regression model, using the formula: E = [10^−1/slope^− 1] × 100% (Chen et al., 2023; Vandesompele et al., 2002; Andersen et al., 2004).

### Total RNA isolation

For each biological sample, it was rapidly ground into a fine powder in liquid nitrogen. Approximately 100 mg of the powdered sample was then weighed, and RNA was extracted using the Trizol reagent. After extraction, the RNA was dissolved in ribonuclease-free water, and the RNA concentration was measured using a ThermoScientific spectrophotometer (SN-1530-801015, Multiskan Sky with Touch Screen). The integrity of the RNA was evaluated by performing 1% agarose gel electrophoresis. The results revealed distinct and intact bands corresponding to the 18S and 28S ribosomal RNA.

Approximately 1 µg of RNA was taken from each sample for cDNA synthesis. First-strand cDNA synthesis was performed using the TransScript All-in-One First-Strand cDNA Synthesis Supermix for qPCR (One-step gDNA removal) (TransGen Biotech, Beijing, China). The synthesized cDNA was diluted four-fold and stored at -20°C for subsequent qRT-PCR.

The gene expression levels in the cDNA samples were evaluated via qRT-PCR. The established qRT-PCR protocol was strictly followed to ensure high-quality RNA for subsequent analysis. The PCR procedure was as follows: 94˚C for 30 s, 42 cycles of 94˚C for 10 s, 60˚C for 30 s, and 72 ˚C for 10 s, with a single melt cycle from 65˚C to 94˚C in 5 s intervals. The Ct values were recorded and further analyses were conducted.

### Reference gene expression stability analysis

The raw fluorescence values obtained from the qRT**-**PCR experiments were imported into Microsoft Excel 2019. Then, the stability of each candidate gene was analyzed using five different methods, namely GeNorm, NormFinder, BestKeeper, and ΔCt (Vandesompele et al., 2002; Andersen et al., 2004; Pfaffl et al., 2004; Silver et al., 2006). Subsequently, RefFinder (Xie et al., 2012, 2023) was combined with ΔCt, GeNorm, NormFinder, and BestKeeper to comprehensively rank the candidate reference genes and assess their stability.

### Validation of reference genes

The expression levels of *SdLDC* (Mancinotti et al., 2022) and a series of related genes involved in regulating seed size and oil synthesis (such as *FAD2* (Al Amin et al., 2019), *MYB73* (Liu et al., 2014), *LEC* (Manan et al., 2017), *NYFA* (Lu et al., 2016), *GA20OX* (Lu et al., 2016), *PP2C* (Lu et al., 2017), *CYP* (Wang et al., 2015), *GPAT* (Hirawake-Mogi et al., 2021) and *PIP* (Lu et al., 2018)) were normalized using single or multiple reference genes to assess the reliability of the selected reference genes during different developmental stages of *S. davidii* seeds. The stability of the reference genes was validated using the 2^-ΔΔct^ method (Livak and Schmittgen, 2001; Ginzinger, 2002). The qRT-PCR amplification conditions were the same as the conditions described above. The primer sequences for the target genes are detailed in **Supplementary Table S1**.

### Statistical analysis for qRT-PCR

Each experiment was performed with three technical replicates. A statistical analysis of variance was carried out on the data obtained from qRT-PCR. With the use of GraphPad Prism 8.3 software (San Diego, California, USA), one-way analysis of variance was employed to test the mean values of each separation at a significance level of 0.05.

The RNA-Seq data used in this study has been uploaded to the NCBI’s GenBank database under the archive number Bankit2870280.

## Results

### Transcriptome data quality analysis

For each developmental stage, cDNA libraries were established with three biological replicates, culminating in a total of 15 libraries. Upon sequencing, each library generated an average of 41,303,713 high-quality clean reads. The Q20 scores exceeded 97%, and the Q30 scores were above 92%. Across all samples, the GC content remained remarkably consistent, ranging from 43% to 46% (**Supplementary Table S2**). Based on these results, it is evident that the transcriptomic data obtained fully meet the requirements for the screening of target genes.

### Verification of primer specificity and PCR efficiency analysis

**Figure 1:**
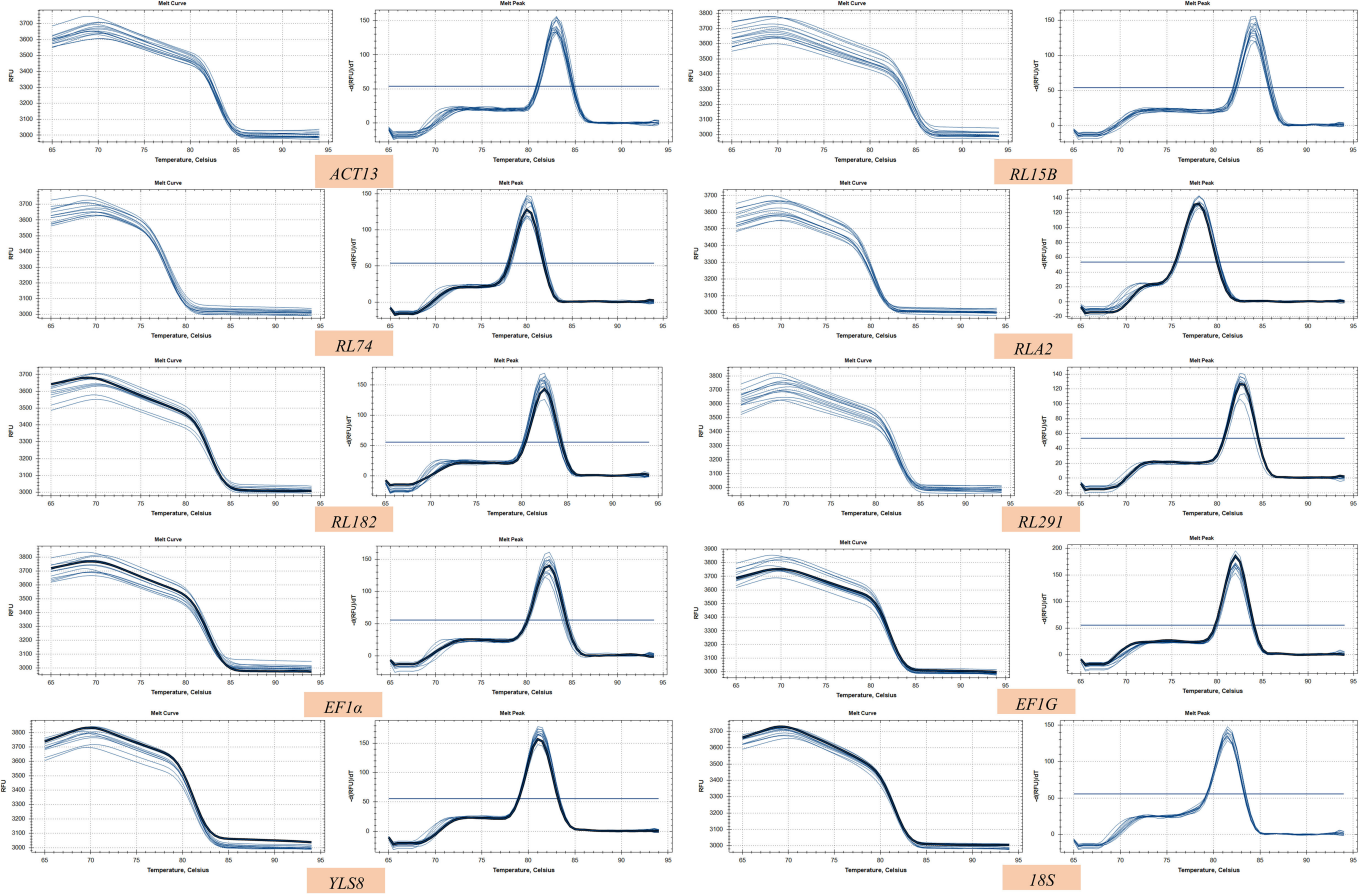
Melting curves of the candidate reference genes and validate genes.

RNA extraction yielded high-quality samples that were well-suited for qPCR analysis (**Supplementary Figure S2**). The standard curve was generated by performing four-fold serial dilutions of the cDNA pool (**Supplementary Figure S3**). The melting curves of all the candidate genes exhibited distinct single peaks (**Figure 1**). The amplification efficiencies of the 10 candidate reference genes ranged from 96.7% to 106.6%, while their correlation coefficients fluctuated between 0.95 and 1 (**Table 1**).

**Table 1:** The candidate reference gene primer sequences and amplicon lengths characteristics.

GENE SYMBOL	GENE DESCRIPTION	GENBANK ACCESSION NUMBER	PRIMER PAIR (5’-3’)	R^2^(%)	EFFICIENCY	AMPLICON LENGTH (BP)	MELTING TEMPERATURE (TM)
*18S*	*18S* RIBOSOMAL RNA	PQ345056	GACTGTGAAACTGCGAATGG TCAAAGCAACAGGCAGAGC	98.75	105.94	105	55.22 55.10
*ACT13*	ACTIN17	PQ345065	GTGTAATGGTTGGGATGGG GTGGTGCCTCGGTAAGAAG	95.00	106.58	203	56.90 54.74
*RL15B*	RIBOSOMAL PROTEIN L15B	PQ345057	TCATTCTGGTGGATGTTGCC CCTGCGGGATGGACGATTT	99.69	96.86	212	54.77 57.48
*RL74*	RIBOSOMAL PROTEIN L74	PQ345058	TTATCCGTATCCGTGGTAT ATGTTCACTGTGGCTTTGT	99.06	102.82	198	50.43 51.90
*RLA2*	RIBOSOMAL PROTEIN LA2	PQ345059	ATCTGGCATGTGCTCCTCT TCAGCCTCAGCTCCAACTA	99.63	103.95	187	56.83 56.68
*RL182*	RIBOSOMAL PROTEIN L182	PQ345060	CGTGTGAAACACTTTGGTCCTG CGGAAATCCTCTACTGTTCCTCCT	99.05	106.97	196	57.28 59.97
*RL291*	RIBOSOMAL PROTEIN L291	PQ345061	CGTAGCCCTATTGGACCTTCA TGTGGCGATGCTTCTTTGG	99.45	100.33	201	57.39 56.94
*EF1G*	EUKARYOTIC TRANSLATION ELONGATION FACTOR 1 GAMMA	PQ345063	GCCTATCTGGTTGGGCATTCTG CTGCTTGACCTGGCCTACT	99.10	97.64	188	59.28 57.33
*YLS8*	YELLOW LEAF-SPECIFIC PROTEIN	PQ345064	GGACAAGCAGGAGTTCATTGACA GTGGAGTAATCTTTGGGAGC	97.85	98.25	145	58.46 58.3
*EF*-*1A*	ELONGATION FACTOR 1-ALPHA, PART OF THE TRANSLATION PROCESS	PQ345062	AACTGGGCTGGCAACTGAA CCCACATTGTCACCAGGAA	99.40	97.47	133	56.46 53.23

### Expression stability of candidate reference genes

10 candidate reference genes were selected for this study. The annealing temperature for primer design ranged from 50.43°C to 59.28°C, and the length of the amplified products varied from 105 bp (corresponding to the *18S* gene) to 212 bp (corresponding to the *RL15B* gene) (**Table 1**). The expressions of these 10 candidate reference genes were assessed by qRT-PCR experiments, with each sample having three technical replicates.

**Figure 2:**
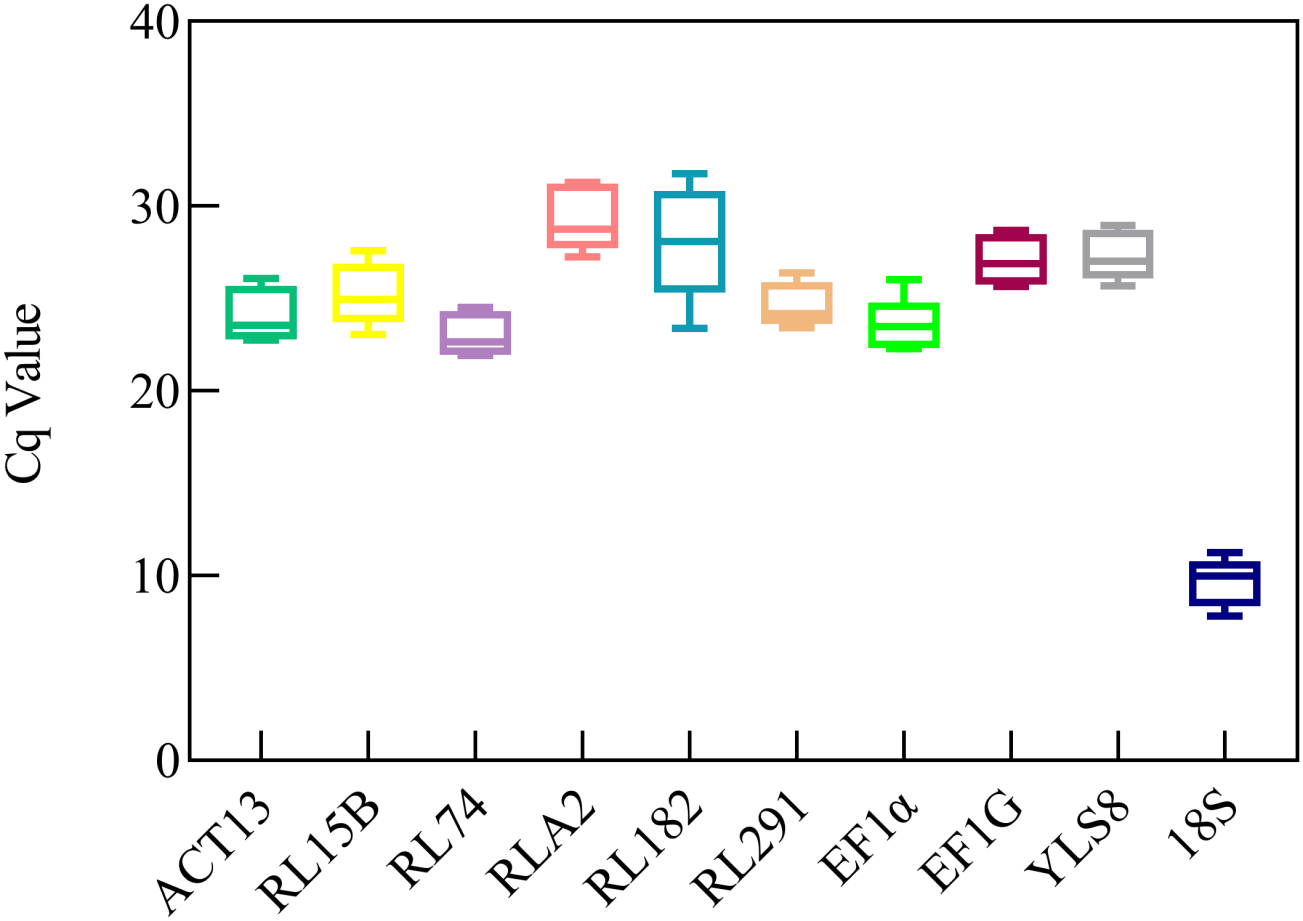
Quantification cycle (Cq) values of tent candidate reference genes in *S. davidii*. A line across the box depicts the median. The box indicates the 25% and 75% percentiles. Whiskers represent the maximum and minimum values. The Figures were generated by using graphpad prism 8.3 software http://www.graphpad.com.

The expression levels of the candidate reference genes were determined based on the Cq values across all samples (**Figure 2**). The average Cq values were distributed between those of *ACT13* and *18S*. Generally, a lower Cq value indicates a higher expression level, whereas a higher Cq value represents a lower expression level (Li et al., 2016). Among all the samples, *18S* demonstrated the highest expression level (9.66 ± 1.33), whereas *RLA2* exhibited the lowest expression level (29.35 ± 1.76). Among the 10 analyzed genes, *RL291* demonstrated a limited range of variation, with expression levels spanning from 23.42 to 26.37. In contrast, *RL182* exhibited the highest degree of variability, with expression levels ranging from 23.38 to 31.76 (**Supplementary Table S3**). The expression profiles of the reference genes were visualized using Ct values presented in a box plot, which enabled an initial evaluation of their stability. The preliminary analysis results suggested that the expression levels of several genes, including *EF*-*1α*, *RL291* were relatively stable and showed high expression levels. However, the coefficients of variation of the *18S* and *RL182* genes reached 13.35% and 10.17%, respectively, suggesting that the expression stability of these two genes was relatively poor among the five samples (**Supplementary Table S3**, **Figure 2**).

### GeNorm analysis

**Figure 3:**
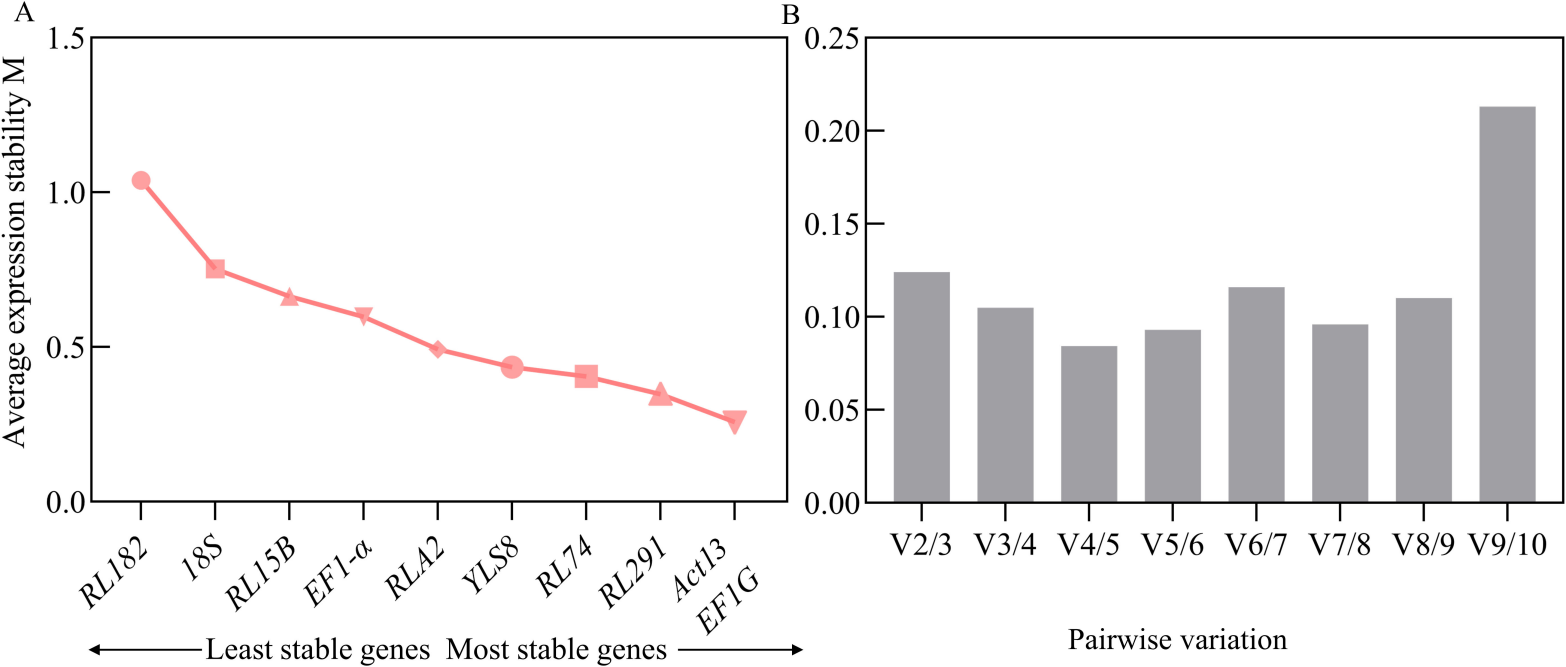
GeNorm software anlysis of the candidate reference genes. **(A)**, Average expression stability values (M) of the candidate reference genes were calculated in *S. davidii* samples. **(B)**, The expression stability and pairwise variation values of ten reference genes obtained, the Pairwise variation (Vn/Vn+1) between the normalization factors was calculated using the geNorm software program to determine the optimal number of candidate reference genes.

The average stability value (M) of the candidate internal reference genes was determined using GeNorm. Generally, a lower M value indicates greater stability of gene expression. A threshold has been set at an M value of less than 1.5 (Vandesompele et al., 2002). The results revealed that the M values of all 10 candidate reference genes were below this threshold, suggesting that their expression was relatively stable and they were preliminarily deemed suitable as reference genes. Based on the analysis by GeNorm, the combination of *ACT13* and *EF1G* exhibited the lowest M value (0.2580), indicating the most stable expression. In contrast, *RL182* presented the highest M value (1.0391), reflecting the least stable expression across different developmental stages of *S. davidii* seeds (**Figure 3A**). To enhance the accuracy and reliability of the evaluation, GeNorm also determines the optimal number of reference genes by analyzing the pairwise differences in the normalization factors of candidate endogenous genes (Vn/Vn+1). When the Vn/Vn+1 value is less than 0.15, it suggests that the selected combination of reference genes is sufficient to achieve stable normalization, thereby ensuring the accuracy and reliability of the experimental outcomes (Vandesompele et al., 2002; Li J. T. et al., 2017). Notably, during the various stages of seed development, the V2/3 value was recorded at 0.124, which was lower than the predefined threshold of 0.15. This finding indicated that the first two genes (*EF1G* and *ACT13*) were sufficient for validation purposes (**Figure 3B**). However, it should be emphasized that the V value mainly serves as a guideline for selecting the optimal number of reference genes, and the threshold of 0.15 is not an absolute critical value. Typically, to achieve more robust normalization, it is recommended to use three reference genes. This approach often yields more reliable results compared to using a single reference gene (Vijayakumar and Sakuntala, 2024; Hu et al., 2023).

### NormFinder analysis

The operational process of NormFinder is comparable to that of GeNorm, as it assesses gene stability by evaluating and quantifying gene expression stability values. Generally, a lower stability value corresponds to higher gene stability (Andersen et al., 2004). When compared to the gene stability ranking generated by GeNorm, the ranking produced by NormFinder showed some variations. Notably, there was a high degree of consistency between the two in terms of the initially identified stable reference genes, with only minor differences in the rankings (**Table 2**). Furthermore, both tools identified *EF1G* as the most stable candidate reference gene, indicating consistency between the two tools.

**Table 2:** *S. davidii* reference genes for normalization and their expression stability order a calculated by GeNorm, Delta CT, NormFinder and Bestkeeper.

Rank	GeNorm		Normfinder		BestKeeper			ΔCT	
	Gene	Stability	Gene	Stability	Gene	SD	CV	gene	SD
1	*EF1G*	0.2580	*EF1G*	0.128	*RL291*	0.99	4.03	*EF1G*	0.72
2	*ACT13*	0.2580	*ACT13*	0.128	*EF1-α*	1.00	4.24	*ACT13*	0.74
3	*RL291*	0.3486	*RL291*	0.346	*RL74*	1.02	4.43	*RL291*	0.80
4	*RL74*	0.4056	*RLA2*	0.362	*18S*	1.04	10.72	*RL74*	0.82
5	*YLS8*	0.4358	*RL74*	0.417	*YLS8*	1.11	4.06	*YLS8*	0.84
6	*RLA2*	0.4933	*YLS8*	0.482	*EF1G*	1.14	4.21	*RLA2*	0.90
7	*EF1-α*	0.5978	*EF1-α*	0.737	*ACT13*	1.28	5.29	*EF1-α*	1.06
8	*RL15B*	0.6650	*RL15B*	0.851	*RL15B*	1.33	5.26	*RL15B*	1.10
9	*18S*	0.7531	*18S*	1.073	*RLA2*	1.49	5.07	*18S*	1.24
10	*RL182*	1.0391	*RL182*	2.126	*RL182*	2.20	7.83	*RL182*	2.18

### Bestkeeper analysis

The BestKeeper software utilizes a dual methodology to evaluate gene stability. Firstly, it calculates the standard deviation (SD) and coefficient of variance (CV) of candidate genes across different samples. Then, these metrics are compared to those of a reference gene, which is selected based on its stability as indicated by its SD and CV values. Genes with SD values below 1.0 are classified as stable and are considered appropriate for normalization in gene expression studies. Specifically, genes having an SD value less than 1.0 are recognized as stable candidates for this normalization process. A lower SD value corresponds to a higher level of stability for the respective gene (Pfaffl et al., 2004). The results indicated that *RL291* (0.99) and *EF1-α* (1.00) were identified as suitable reference genes in the seeds of *S. davidii* at various developmental stages (**Table 2**).

### ΔCT analysis

The ΔCT method assesses gene expression stability by calculating the SD and CV of Ct values. Generally, lower SD and CV indicate higher expression stability (Silver et al., 2006). The results revealed that *EF1G* demonstrated the lowest SD value, suggesting the highest expression stability. Conversely, *RL182* had the highest SD value, making it the most unstable gene (**Table 2**). The expression stability of the 10 candidate genes exhibited minimal variation among the statistical methods that were used. All four analytical software programs identified *RL182* as the most unstable candidate gene for the *S. davidii* seed at various developmental stages.

### Refinder analysis

ReFinder is a web-based evaluation tool that integrates the results of the previous four methods for ranking and reaches consistent results (Xie et al., 2012, 2023). This tool computes the geometric mean of each reference gene according to the rankings from four statistical methods. Generally, a lower ranking value implies higher expression stability. The results indicated that *EF1G* had the lowest ranking value. It was followed by *RL291*, *ACT13*, *RL74*, *EF1-α*, *YLS8*, *RLA2*, *18S*, *RL15B*, and *RL182*. Among them, *RL182* had the highest rank value (**Table 3**).

**Table 3:** ReFinder ranking of 10 candidate reference genes.

Rank	Candidate genes	Ranking value
1	*EF1G*	1.57
2	*RL291*	2.28
3	*ACT13*	2.30
4	*RL74*	3.94
5	*EF1-a*	5.12
6	*YLS8*	5.23
7	*RLA2*	6.00
8	*18S*	7.35
9	*RL15B*	8.00
10	*RL182*	10.00

**Figure 4:**
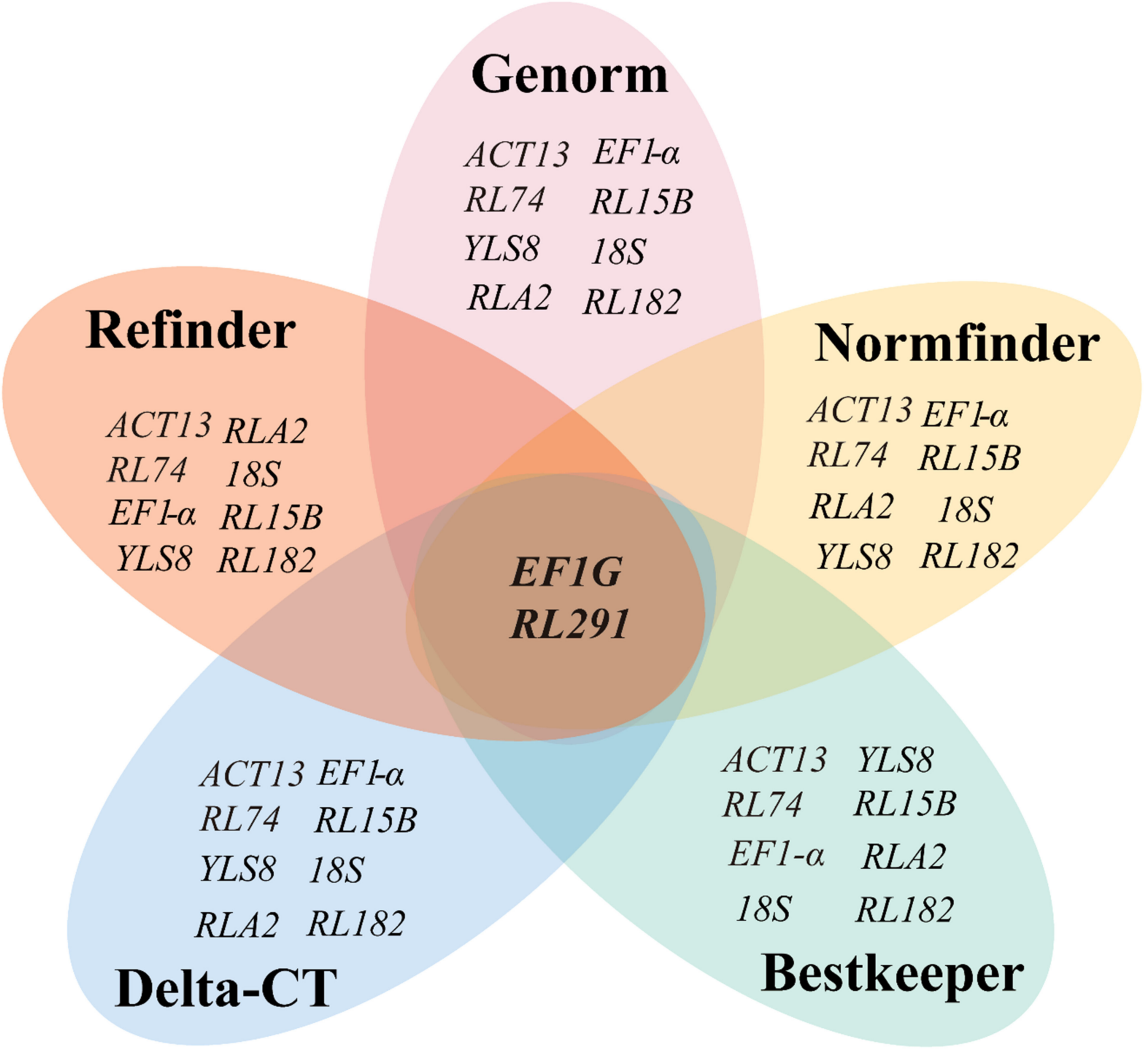
Venn diagram showing the most stable reference genes identified by the GeNorm, NormFinder, BestKeeper, ΔCT, and RefFinder algorithms. The intersection part reveals the most stable genes that are common among them, specifically *EF1G* and *RL291*. The mapping data were derived from **Tables 2**, **3**.

**Figure 5:**
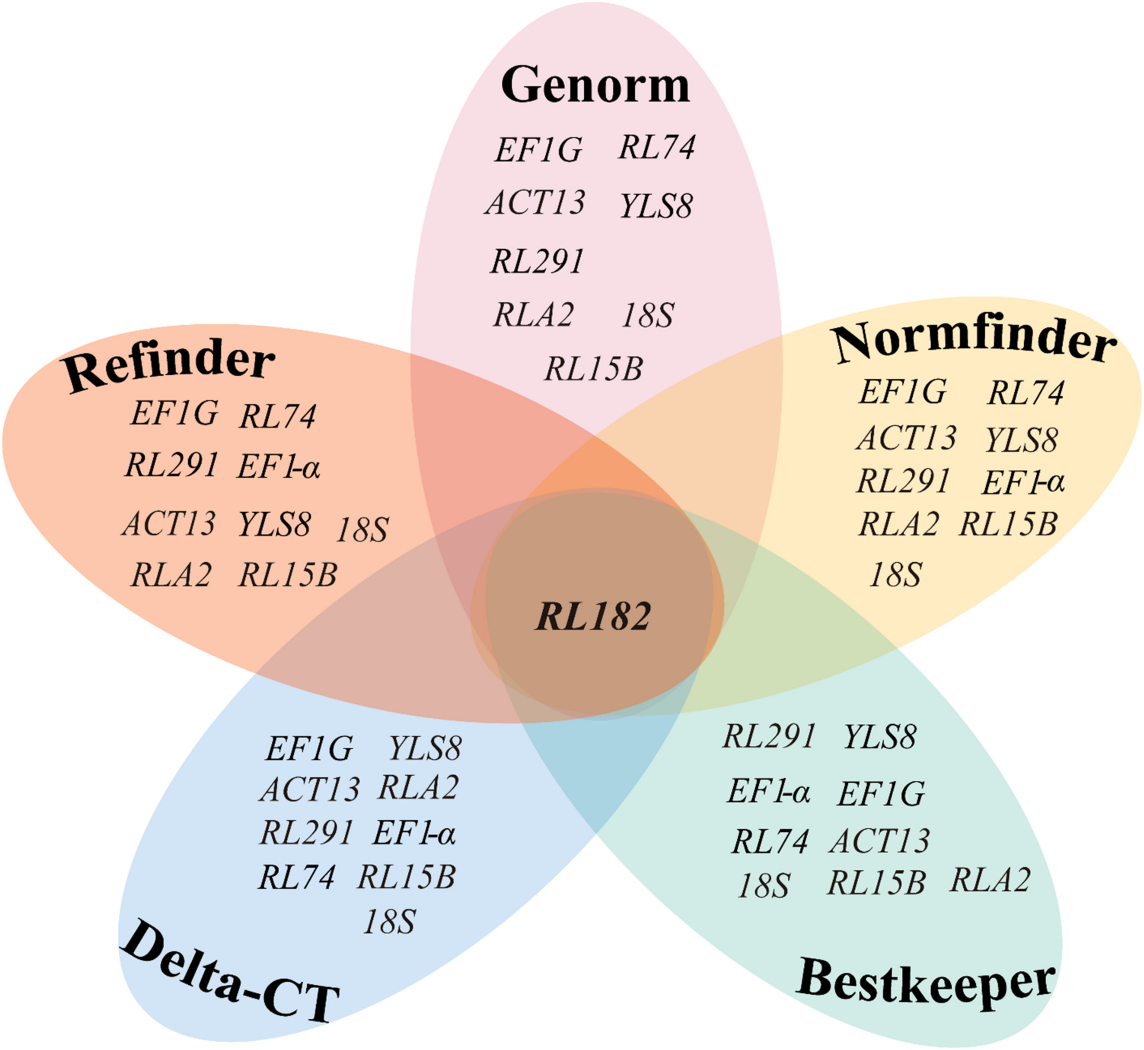
Venn diagram showing the least stable reference genes identified by the GeNorm, NormFinder, BestKeeper, ΔCT, and RefFinder algorithms. The intersection part reveals the most stable genes that are common among them, specifically *EF1G* and *RL291*. The mapping data were derived from **Tables 2**, **3**.

Overall, these five algorithms yielded various results when selecting the optimal reference genes for different developmental stages of seeds in *S. davidii*. Generally speaking, *EF1G* and *RL291* exhibited the highest expression stability and were suitable as reference genes (**Figure 4**), whereas *RL182* was the most unstable gene (**Figure 5**).

### Validation of the stability of reference genes

**Figure 6:**
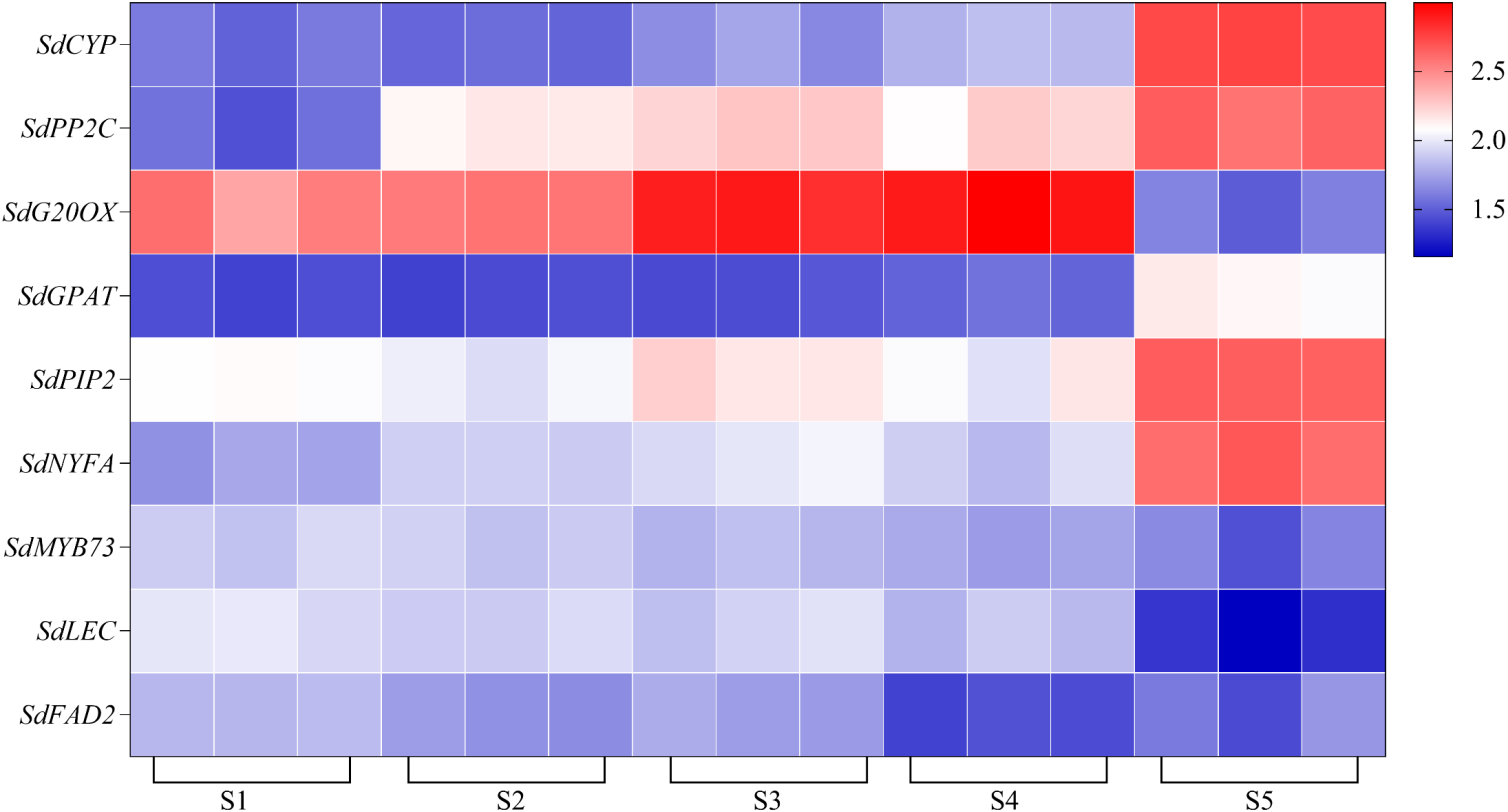
Heat map of the expression levels of selected key genes as likely to be involved in oil content accumulation and grain formation (*SdCYP*, *SdFAD2*, *SdG20OX*, *SdGPAT*, *SdLEC*, *SdNYFA*, *SdPP2C*, *SdMYB73*, *SdPIP2*).

The results from the five algorithms indicated that the expressions of *EF1G* and *RL291* were relatively stable. To validate the appropriateness of the identified candidate reference genes, we compared the expression patterns of target genes in the samples using FPKM-normalized RNA-seq data (**Figure 6**). Additionally, we confirmed these findings through a comprehensive analysis using qRT-PCR normalization.

**Figure 7:**
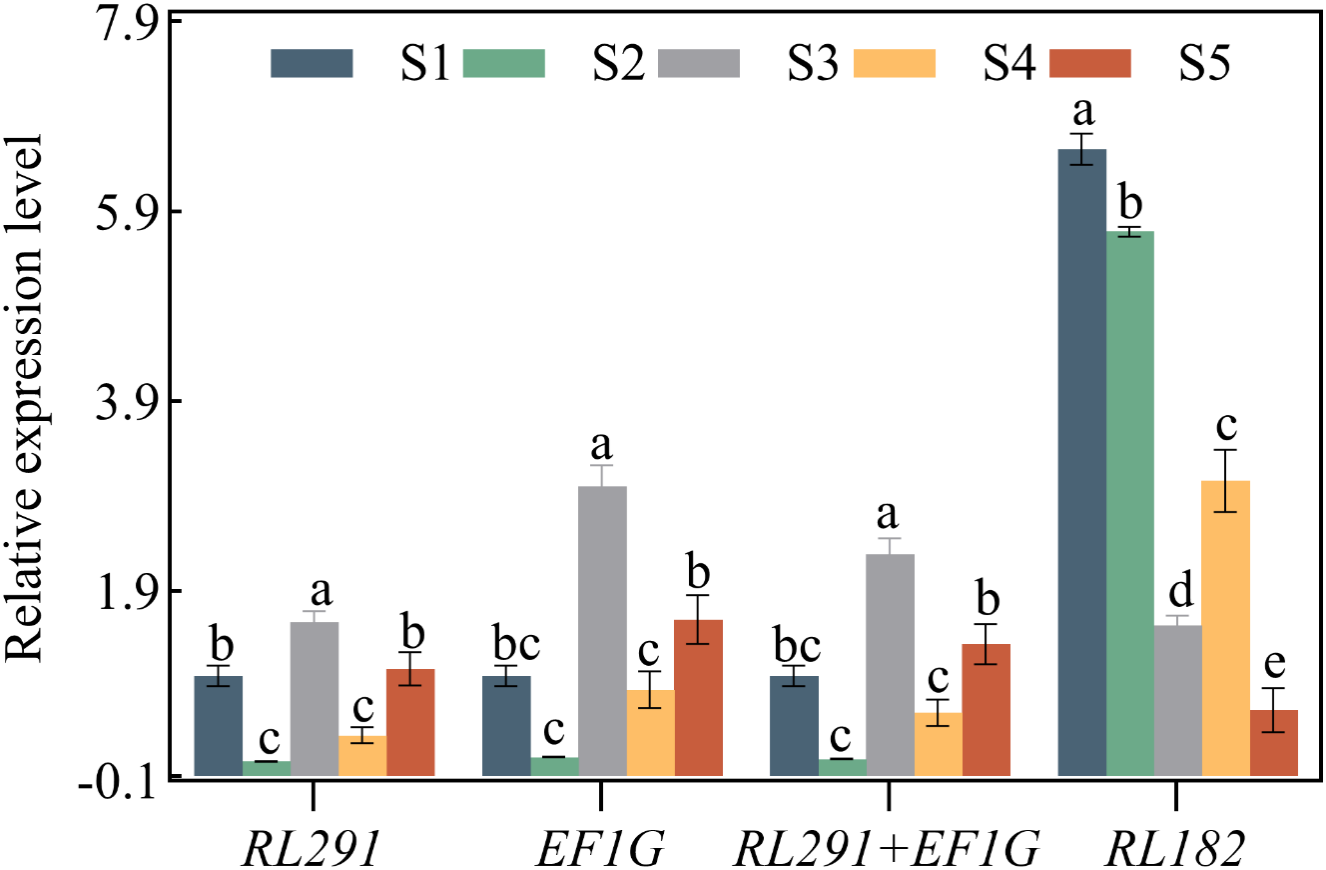
Validation of the identified reference genes of key genes *SdLDC*. Expression levels of *SdLDC* in different seed development stages of *S. davidii*. The expression data were normalized using the most stable reference genes (*EF1G*, *RL291*, and *RL291*+*EF1G*) and the least stable reference gene (*RL182*), respectively. The x-axis represents the comparison between the final selection of the most stable reference genes and the least stable reference gene (*RL182*) in the seed organ of *S. davidii*. Data were compared and analyzed using analysis of variance and multiple comparisons with the statistical analysis software GraphPad Prism 8.3. P<0.05 was considered to indicate a statistically significant difference.

**Figure 8:**
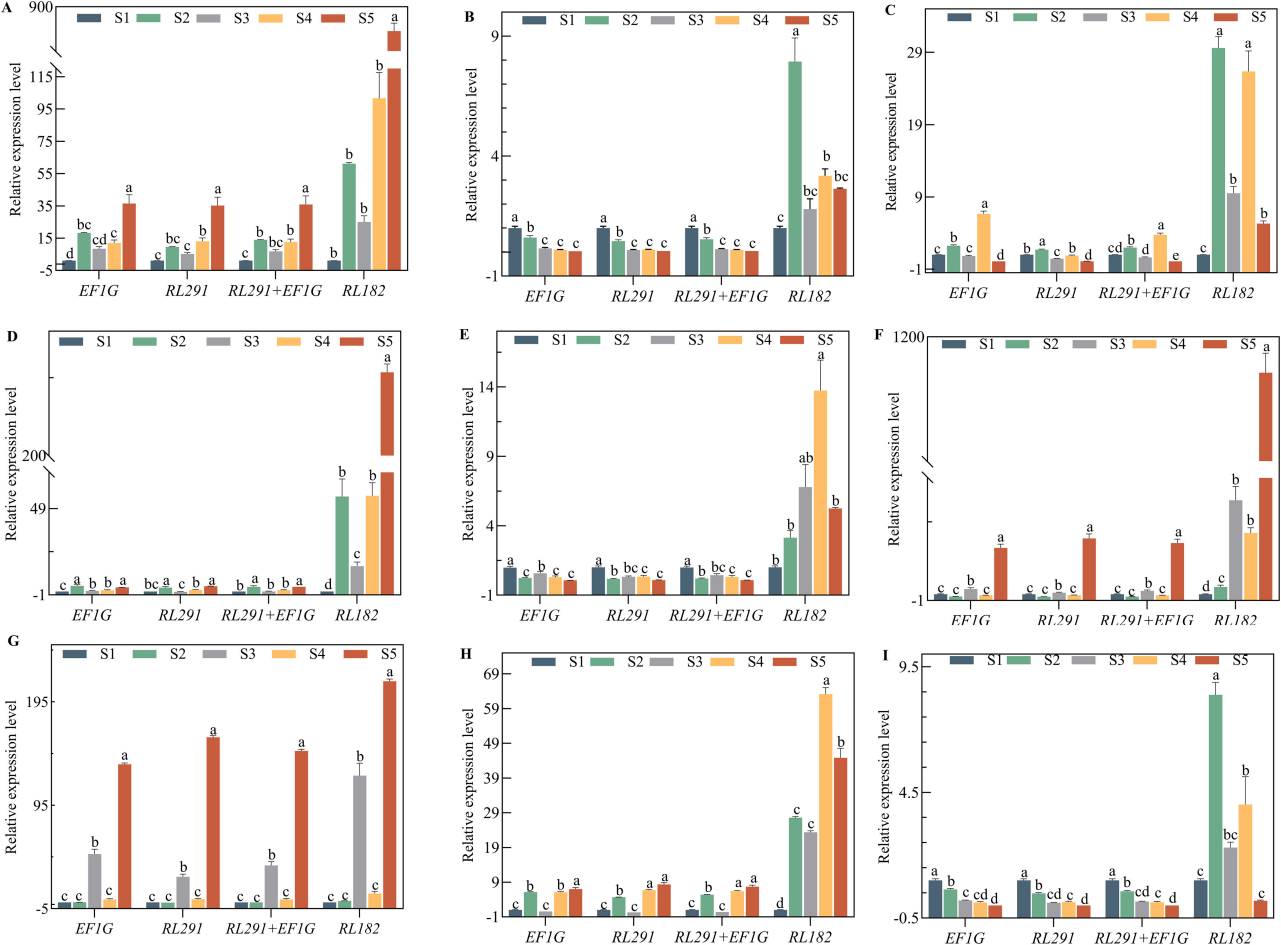
Validation of the identified reference genes of key genes as likely to be involved in oil content accumulation and grain formation. Expression levels of **(A)** *SdCYP*, **(B)** *SdFDA2*, **(C)** *SdG20X1*, **(D)** *SdGPAT*, **(E)** *SdLEC*, **(F)** *SdNYFA*, **(G)** *SdPP2C*, **(H)** *SdMYB*, and **(I)** *SdPIP* in different seed development stages of *S. davidii*. The expression data were normalized using the most stable reference genes (*EF1G*, *RL291*, and *RL291*+*EF1G*) and the least stable reference gene (*RL182*), respectively. The x-axis represents the comparison between the final selection of the most stable reference genes and the least stable reference gene (*RL182*) in the seed organ of *S. davidii*. Data were compared and analyzed using analysis of variance and multiple comparisons with the statistical analysis software GraphPad Prism 8.3. P<0.05 was considered to indicate a statistically significant difference.

The expression profiles of *SdLDC* (a functional gene related to quinsolizidine alkaloid biosynthesis) and related genes that regulate seed size and oil synthesis (*FAD2*, *MYB73*, *LEC*, *NYFA*, *GA20OX*, *PP2C*, *CYP*, *WRKY15a*, *PIP*) were normalized using single or multiple reference genes. The reference genes included *RL291* and *EF1G*, either used alone or in combination, as well as the least stable internal reference gene *RL182*. This was done to test the reliability of the selected reference genes at different developmental stages of *S. davidii* seeds (**Figures 7**, **8**). In this study, we selected the top two most stable and the least stable reference genes at different stages for normalization, combined as *EF1G* and *RL291*, for different development stages of seeds. There were variations in the expression patterns of these 10 target genes in the seeds. After standardizing the data with the top two reference genes, the expression patterns of the 10 target genes were consistent. When normalized using the lowest-ranked reference gene (*RL291*), the expression of the target gene was significantly higher than when using the most stable gene. The reference genes with the lowest rankings were not optimal for data normalization. When using these genes, the expression levels of target genes varied significantly compared to those obtained with the top-ranked reference genes. These results clearly indicated that the use of inappropriate reference genes could lead to misleading results in the normalization of target genes. Therefore, the results of our analysis further underscored the necessity of selecting reference genes with appropriate stability before conducting q-RT PCR studies to avoid low accuracy. In conclusion, the combination of *RL291* and *EF1G* represents the optimal set of reference genes for the normalization of qRT-PCR data during the developmental stages of *S. davidii* seeds.

## Discussion

### The critical role of reference genes in qRT-PCR normalization

Gene expression analysis is a fundamental approach for investigating regulatory pathways and metabolic activities within living organisms. qRT-PCR is a powerful technique extensively utilized in gene expression studies across a wide range of organisms, including plants, animals, and microorganisms (Zhao et al., 2024; Deepak et al., 2007; Limothai et al., 2020; Shawon et al., 2024; Yi et al., 2022; Muñoz et al., 2021). Many studies have demonstrated that no single reference gene can be effectively employed for qPCR across all species or experimental conditions. The use of stably expressed reference genes is of utmost importance for precise standardization. This practice can mitigate technical variability and ensure reliable quantification of target transcripts (França and Cerca, 2023; Liu et al., 2018). Although conventional housekeeping genes, such as *actin* and *tubulin*, are widely used, their expression stability is context-dependent and species-specific. This has been verified through studies on bacterial biofilms and plant stress responses (Zhang et al., 2022; Thellin et al., 1999). For example, Leng et al. discovered in pan-cancer platelet research that *GAPDH* was stably expressed in both cancerous and healthy samples, making it an ideal reference gene (Wen et al., 2022). Nevertheless, under calcium stress, the stable expression of *GAPDH* in HA-1 cells was disrupted, introducing systematic errors in the quantification of target genes (Suzuki et al., 2018). Vorachek et al. (2013) demonstrated that canonical reference genes, like *PGK1*, *ACTB*, and *B2M*, exhibited unstable expressions when neutrophils were infected, inflamed, or under stress. This poses a challenge for reliable gene expression normalization. An incorrect selection of reference genes can lead to inaccurate gene expression results (Guénin et al., 2009; Chen et al., 2019). Therefore, appropriate reference genes are the cornerstone for accurate quantification in qRT-PCR experiments, and their stability must be experimentally verified under specific conditions. In this study, improper standardization using unstable genes like *RL182* led to significant variations in the expression profile of *SdLDC*, underscoring the necessity of rigorous validation.

### Methods for evaluating reference gene stability

To minimize the bias in gene expression quantification of *S. davidii*, this study employed various mathematical and statistical models. Current methods for evaluating the stability of reference genes include the ΔCt, GeNorm, NormFinder, BestKeeper, and RefFinder. ΔCt assesses stability by calculating the difference in Ct values between candidate and target genes. A smaller difference indicates higher expression consistency (Silver et al., 2006). GeNorm uses pairwise comparison to compute the average logarithmic variation coefficient (M value). Genes with M values less than 1.5 are generally considered stable (Vandesompele et al., 2002). NormFinder applies analysis of variance to simulate intra- and inter-group expression variability and generates stability values for ranking reference gene candidates (Andersen et al., 2004). BestKeeper evaluates gene stability by analyzing the SD of Ct values and Pearson correlation (r) (Pfaffl et al., 2004). RefFinder integrates multiple algorithm results to produce a composite ranking, reducing method-specific bias (Xie et al., 2023). These tools evaluate stability from various angles, such as expression discrepancy, coefficient of variation, analysis of variance, and statistical correlation. Combining them can enhance the reliability of reference gene validation. However, these algorithms are not suitable for all experiments. For example, GeNorm is typically used when there are three or more candidate genes. Its results are affected by sample type and processing conditions (Vandesompele et al., 2002). Gene co-regulation may also interfere with the analysis because the software tends to select genes with similar expression profiles (Andersen et al., 2004). Therefore, it is necessary to choose an appropriate algorithm based on the experimental objectives. Among the candidate genes in this study, *EF1G* and *RL291* ranked highly in all algorithms and showed the most stable expression. Regardless of the calculation method, *RL182* was identified as the most unstable reference gene (**Figures 4**, **5**). This highlights the importance of using multiple approaches to evaluate reference gene stability, ensuring that only the most reliable genes are selected for normalization.

### The importance of RNA-seq in reference gene selection and gene expression analysis

RNA-seq is fundamental for understanding gene function and structure. It also plays a pivotal role in uncovering biological development and pathological mechanisms. Transcriptomics can help identify disease biomarkers, decipher signaling pathways, and determine therapeutic targets by comprehensively capturing dynamic gene expression patterns (Wang et al., 2009). By conducting an in-depth analysis of the whole transcriptome, researchers can identify genes that are stably expressed under specific biological conditions. This is of great significance for the selection of reliable reference genes. For instance, RNA-seq technology enabled researchers to discover the splicing pattern of introns in the organelle genome of *Nymphaea* species, providing new insights for reference gene selection (He et al., 2021).

In this study, we utilized multiple normalization factors, including individual genes like *EF1α*, *RL291*, *RL182*, and the *EF1α-RL291* gene combination, to normalize the expression of several validation genes. Although the absolute values of gene abundance varied among different normalization methods, the gene expression patterns at different stages of seed development (depicted by the log-transformed FPKM values of RNA-seq) were highly consistent (**Figures 6**–**8**). Notably, using a multi-gene combination for normalization significantly improved the accuracy of the quantitative results. Conversely, using the most unstable gene, *RL182*, as a reference led to significant differences in the expression pattern of the validation gene (**Figures 7**, **8**).

### The significance of key gene expression analysis in biosynthetic pathway elucidation

In plant research, selecting the most appropriate reference genes is crucial for understanding gene expression and metabolic pathway regulation (Gutierrez et al., 2008). Take *S. davidii* for example. Its roots, leaves, flowers, and fruits are well-known for their medicinal properties. In particular, quinolizidine alkaloids like sophoridine and oxymatrine are present, which have potential therapeutic applications for various cancers (Rashid et al., 2019). However, the limited production of bioactive compounds restricts the widespread use of these plants in traditional medicine. This highlights the need to explore genetic resources for secondary metabolite biosynthesis. Although *S. davidii* has significant therapeutic value, little is currently known about its molecular mechanisms. Most quinolizidine alkaloids reach their highest concentration in seeds (Gotti and Clementi, 2021). Research revealed that the alkaloid content in *S. davidii* seeds was higher than in other tissues such as leaves and flowers (Xu et al., 2023). Therefore, in this study, samples were collected from key stages of seed development in *S. davidii* to investigate the expression patterns of key genes and determine the spatial specificity of enzyme-catalyzed reactions in biosynthetic pathways. The results showed that LDC, the first key enzyme in alkaloid biosynthesis, exhibited higher expression levels at the S3 stage (**Figure 7**). During the seed maturation stage, LDC catalyzes the decarboxylation of lysine to produce alkaloids, which play a crucial role in plant defense mechanisms and seed protection (Yang et al., 2016). The possible reason for this phenomenon is that during the seed maturation stage, LDC catalyzes the decarboxylation of lysine to produce alkaloids. These alkaloids play a vital role in the plant’s defense mechanisms and the protection of its seeds. Consequently, the high expression of LDC when the seeds are fully developed meets the demand for alkaloid synthesis.

## Conclusion

In qRT-PCR, the selection of standardized and reliable internal controls is often neglected, yet it is a critical factor directly influencing the rigor of gene expression analysis. Although qRT-PCR is widely used for quantifying relative gene expression, the accuracy of results may decline if stable reference genes are not carefully chosen, thereby undermining data reliability. Therefore, identifying and validating appropriate reference genes is essential to ensure the accuracy of gene expression measurements.

This study aimed to identify reliable reference genes for qRT-PCR of *S. davidii* seeds, which is significant for understanding quinolizidine alkaloid biosynthesis. By conducting transcriptome-guided screening and validating with multiple algorithms (GeNorm, NormFinder, BestKeeper, ΔCT, and RefFinder), we identified that *EF1G* and *RL291* are the optimal reference genes for normalizing qRT-PCR data at five distinct morphological stages of seed development. The reliability of these genes was verified through qRT-PCR and various analytical methods. These findings provide a solid foundation for accurate gene expression analysis of *S. davidii*, particularly in investigating alkaloid biosynthetic pathways. This research underscores the importance of transcriptome sequencing in selecting the optimal reference genes and paves the way for further research on key enzymes in the alkaloid biosynthesis of *S. davidii*.

## Data Availability

The raw data supporting the conclusions of this article will be made available by the authors, without undue reservation.
