# Supplementary material for: Selection and validation of reference genes for quantitative real-time PCR during the developmental stages of seeds in Sophora davidii
Source: Front Plant Sci. 2025 May 16;16:1485586. doi: 10.3389/fpls.2025.1485586 (PMC12122454; doi:10.3389/fpls.2025.1485586)
Supplement: Supplementary file 1 [file DataSheet1.docx]

**Selection and Validation of Reference Genes for Quantitative Real-Time PCR During the Developmental Stages of Seeds in in Sophora davidii**

**The running title: Reference Genes for Quantitative Real-Time PCR in *Sophora davidiii***

Jingjing Li ^1,2^, Shanrong Han^1,2^, Zongren Xu^2^, Bin Deng^3^, Hong Zhang^2^, Zongsuo Liang^2^, Jing Liu^2*^, Shua Liu ^2^***,** Na Zheng^1,2^, Yaqiong Su^1,2^，Ziyao Qiao^1,2^ and Yun Yang^1,2^

^1^ Key Laboratory of Resource Biology and Biotechnology in Western China, Ministry of Education/College of Life Science, Northwest University, Xi’an, Shaanxi 710069, China

^2^ Shaanxi Academy of Traditional Chinese Medicine, Xi'an, Shaanxi 710003, China

^3^ School of Pharmacy, Guizhou University of Traditional Chinese Medicine, Guiyang, China

* Correspondence: Shuai Liu (email: [Shuai_Liu89@63.com](mailto:yltang@szu.edu.cn))

Email addresses of all authors:

Jingjing Li (ddap0091@163.com); Zongren Xu (xuzongren1125@163.com); Bin Deng (572962400@qq.com); Shanrong Han (3050802591@qq.com); Hong Zhang (zhanghong919919@163.com); Zongsuo Liang (liangzs@zstu.edu.cn); Jing Liu ([Stardustj@163.com](mailto:Stardustj@163.com)); Na Zheng (18700967692@163.com); Yaqiong Su (suyaqiong2022@163.com)，Ziyao Qiao ([qiaoziyao0501@163.com](mailto:qiaoziyao0501@163.com)); Yun Yang (y2ngyun651@163.com)

**
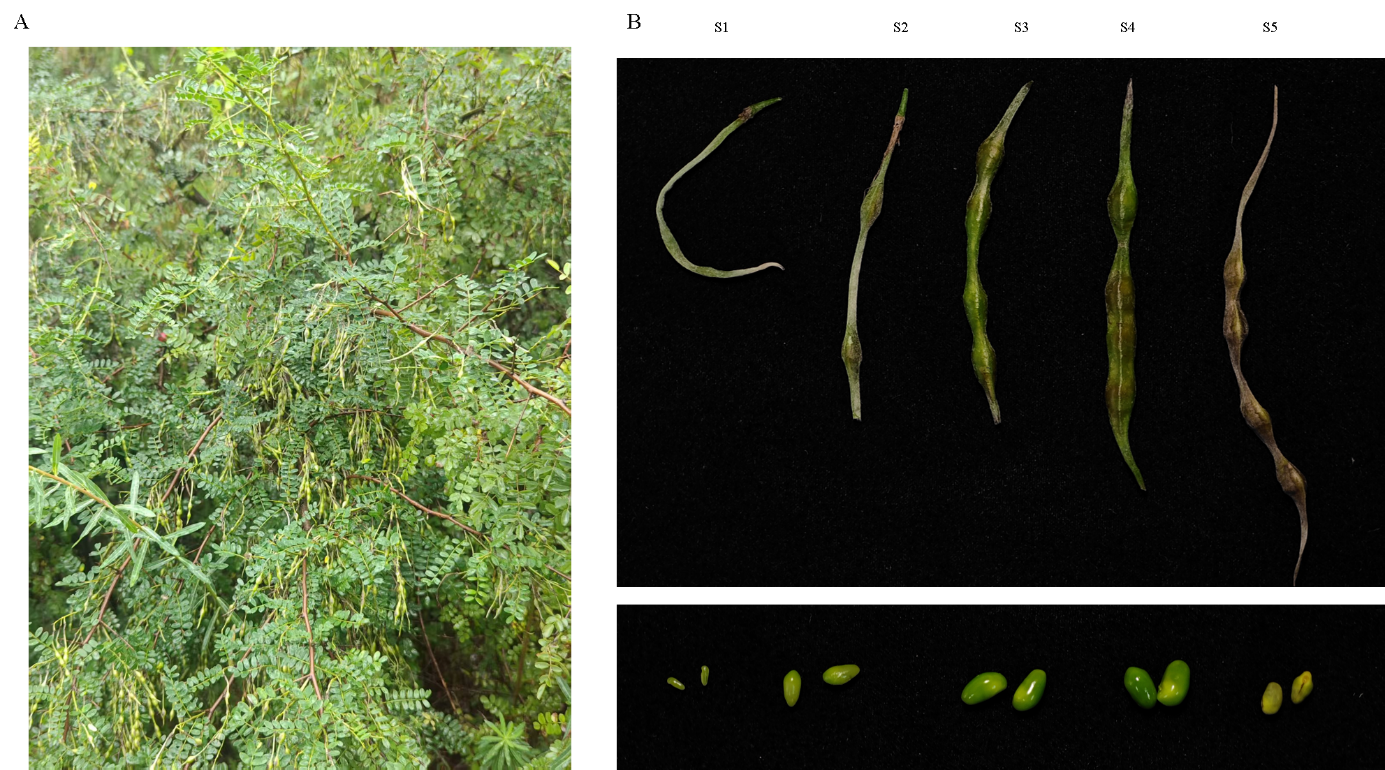
**

**Figure S1 Morphological diagram of *S. davidii***

A: Illustration of the Pods Hanging on the Tree; B: The five-stage development of Seeds of *S. davidii*


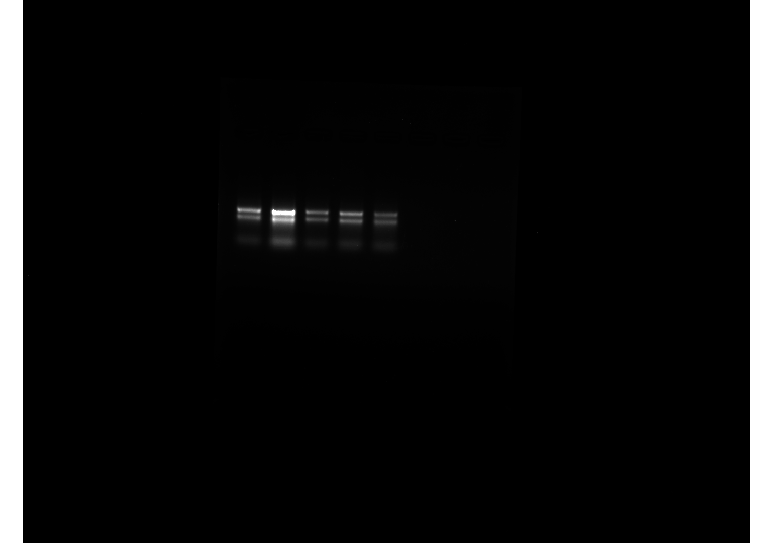


S1 S2 S3 S4 S5

Figure.S2 Isolation of RNA from from *S. davidii* seeds at different developmental stages

RNA integrity was analyzed by 1% agarose gel electrophoresis, two clear and complete bands, 18S and 28S, can be seen on the nucleic acid gel and the intensity of the 28S rRNA band should be roughly twice that of the 18S rRNA band, which can be used for the subsequent.

The RNA concentration and purity from S1 to S5 are as follows in sequence.

| Sample | S1 | S2 | S3 | S4 | S5 |
| --- | --- | --- | --- | --- | --- |
| Concentration (μg/ml)  Quality | 611 | 883 | 412 | 400 | 367 |
| OD260/230 | 2.19 | 2.20 | 2.04 | 2.04 | 2.03 |
| OD280/260 | 1.85 | 1.99 | 1.87 | 1.80 | 1.86 |

**
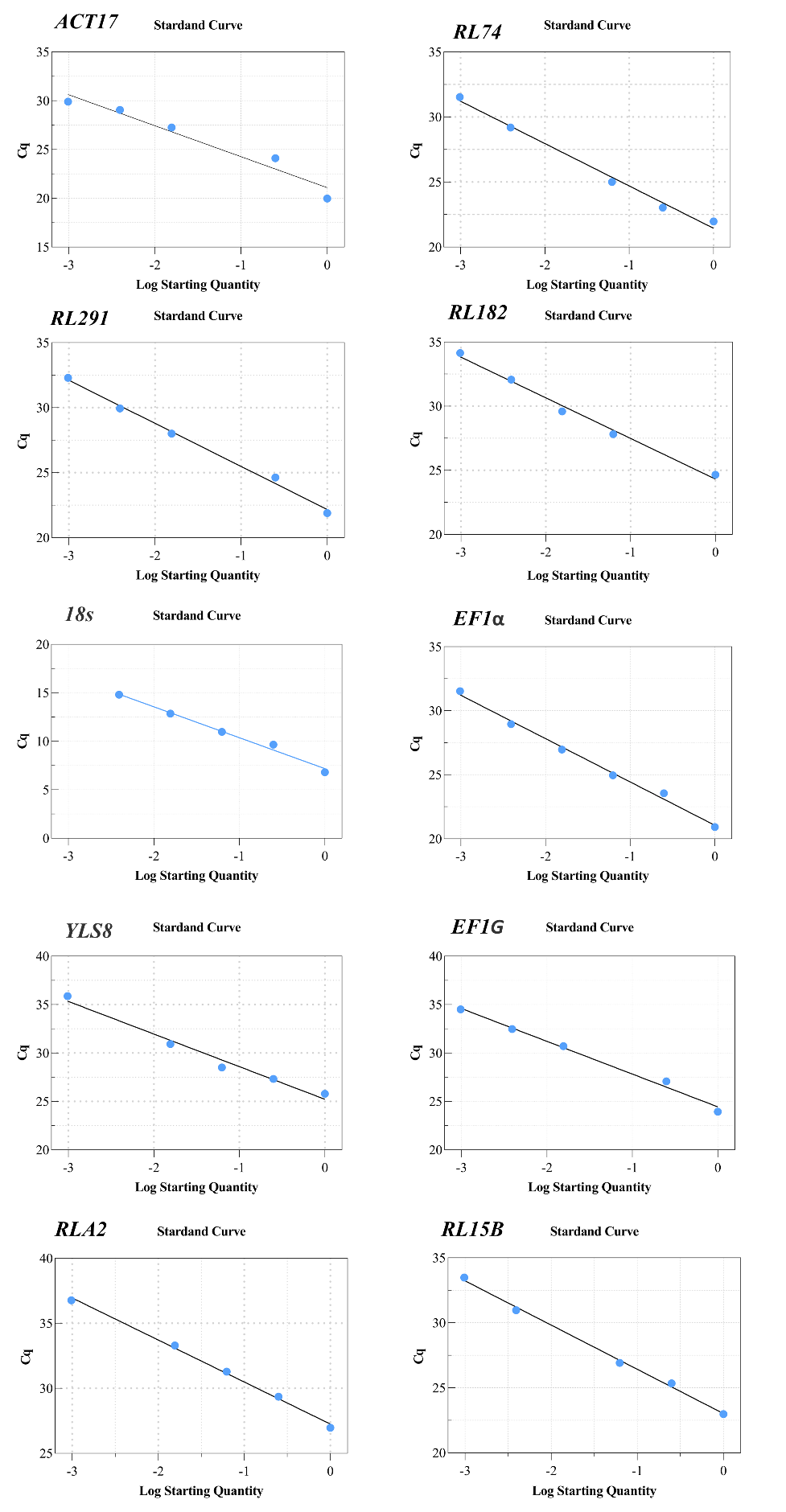
**

Figure S3. Standard curves of each candidate genes in *S. david*
